# School environment associates with lung function and autonomic nervous system activity in children: a cross-sectional study

**DOI:** 10.1038/s41598-019-51659-y

**Published:** 2019-10-22

**Authors:** Inês Paciência, João Cavaleiro Rufo, Diana Silva, Carla Martins, Francisca Mendes, Tiago Rama, Ana Rodolfo, Joana Madureira, Luís Delgado, Eduardo de Oliveira Fernandes, Patrícia Padrão, Pedro Moreira, Milton Severo, Maria Fátima Pina, João Paulo Teixeira, Henrique Barros, Lasse Ruokolainen, Tari Haahtela, André Moreira

**Affiliations:** 1 Faculdade de Medicina da Universidade do Porto, Porto, Portugal & Centro Hospitalar São João, Porto, Portugal; 2 Institute of Science and Innovation in Mechanical Engineering and Industrial Management (INEGI), Porto, Portugal; 3 EPIUnit - Instituto de Saúde Pública, Universidade do Porto, Porto, Portugal; 4 Faculdade de Ciências da Nutrição e Alimentação da Universidade do Porto, Porto, Portugal; 5 Departamento de Epidemiologia Clínica, Medicina Preditiva e Saúde Pública da Faculdade de Medicina da Universidade do Porto, Porto, Portugal; 6 Instituto de Investigação e Inovação em Saúde (I3S), Universidade do Porto, Porto, Portugal; 7 Instituto de Engenharia Biomédica (INEB), Universidade do Porto, Porto, Portugal; 8 Health Communication and Information Institute, Fundação Oswaldo Cruz (ICICT/FIOCRUZ), Rio de Janeiro, Brazil; 9 Environmental Health Department, Portuguese National Institute of Health, Porto, Portugal; 10 Department of Biosciences, University of Helsinki, Helsinki, Finland; 11 Skin and Allergy Hospital, Helsinki University Central Hospital, Helsinki, Finland

**Keywords:** Environmental impact, Risk factors

## Abstract

Children are in contact with local environments, which may affect respiratory symptoms and allergic sensitization. We aimed to assess the effect of the environment and the walkability surrounding schools on lung function, airway inflammation and autonomic nervous system activity. Data on 701 children from 20 primary schools were analysed. Lung function, airway inflammation and pH from exhaled breath condensate were measured. Pupillometry was performed to evaluate autonomic activity. Land use composition and walkability index were quantified within a 500 m buffer zone around schools. The proportion of effects explained by the school environment was measured by mixed-effect models. We found that green school areas tended to be associated with higher lung volumes (FVC, FEV1 and FEF25–75%) compared with built areas. FVC was significantly lower in-built than in green areas. After adjustment, the school environment explained 23%, 34% and 99.9% of the school effect on FVC, FEV1, and FEF25–75%, respectively. The walkability of school neighbourhoods was negatively associated with both pupil constriction amplitude and redilatation time, explaining −16% to 18% of parasympathetic and 8% to 29% of sympathetic activity. Our findings suggest that the environment surrounding schools has an effect on the lung function of its students. This effect may be partially mediated by the autonomic nervous system.

## Introduction

Urbanization is one of the leading global trends of the 21^st^ century, with significant changes in living standards, lifestyles, social behaviour, and health. Steady urbanization has increased the relevance of understanding the relationships between the environment and human health and wellbeing. While an increased standard of living offers many opportunities, unhealthy diets, physical inactivity, and exposure to urban air pollution are unfortunate side effects of urbanization^1^.

Over the past decades, urbanization and the Western lifestyle have been linked to the rising prevalence of inflammatory disorders, including asthma and allergic diseases. Epidemiological studies have demonstrated that several urban factors, such as traffic-related air pollution, residential proximity to roads and heavy traffic, and household characteristics, are associated with reduced lung function^2,3^ and increased risk of asthma-related symptoms^4,5^. However, the pathways whereby they influence lung function and the development of asthma are complex and interactive. One of the possible mechanisms is the induction of a persistent inflammatory state mediated by the immune system^6^. Airway inflammation is an important factor in the pathogenesis and pathophysiology of asthma. The dysregulation of endogenous immune processes, particularly by the autonomic nervous system, are, in part, responsible for the development and chronicity of asthma^7^.

The human airways are innervated by efferent and afferent autonomic nerves, which regulate many aspects of airway physiology, including airway smooth muscle tone, mucus secretion, microvascular permeability, and the recruitment and activation of inflammatory cells^8,9^. The parasympathetic nervous system is the dominant neuronal pathway in the control of smooth muscle tone and secretion in airways^8^. In asthmatics patients, increased basal parasympathetic tone is observed^10,11^. This results in constricted airways and an enhanced bronchoconstriction response to different inhaled agents that are known to stimulate airway C-fibre sensory nerves^12,13^. In turn, their activation due to environmental exposure may lead to the release of neuropeptides locally by transient receptor potential (TRP) cation channels, resulting in cough, airway irritation, mucous secretion, and bronchoconstriction mediated by the efferent pathways of the autonomic nervous system^14,15^. Nevertheless, these mechanisms are associated not only with urban factors but also with individual determinants and behaviours, such as physical activity, diet, and obesity^16,17^.

The complexity of the interactions among urbanization, environmental change and human health and wellbeing requires an integrated approach. Therefore, to be effective in promoting health and healthy behaviour, public health interventions should address not only individual characteristics but also the physical and social environment^1^. A few studies have focused on the relationship between the surrounding greenness levels in children’s living environment and their health^18–20^. However, since children spend a large proportion of their time at school, the school environment has recently garnered attention as a potential contributor to child health^21^. Thus, the aim of the present study was to evaluate the effect of school neighbourhoods and their walkability on lung function, airway inflammation and autonomic nervous system activity in children.

## Results

An increased proportion of built areas in the school neighbourhood was associated with significantly lower values of FVC (model 0: β = −5.13, 95% CI −9.36, −0.91; model 2: β = −4.98, 95% CI −10.3, −0.35), while green areas showed a tendency to be associated with higher values of FVC, FEV_1_ and FEF_25–75%_ (Supplementary Table S1). The highest ICCs were observed for FEV_1_ and FVC (0.40% and 0.04%, respectively), indicating that approximately 1% of the total variation in these parameters was found between schools. After adjustment for age, sex, asthma, WHO z-score for BMI and family history of asthma or allergy, the neighbouring environment explained 98%, 96%, and >99.9% of the effect of school on FVC, FEV_1_, and FEF_25–75%_, respectively (model 5, Table 1; Supplementary Fig. S1). No associations were observed between school neighbourhood and EBC pH (Supplementary Fig. S2) or exhaled NO (Supplementary Fig. S3).

**Table 1:** Multilevel model analysis of the association between individual and neighbouring environment and lung function, pH, exhaled NO and pupillometry parameters explained by school.

Outcome	β (95% CI)		School		
			ICC	Variance	Explained variation*
	PC1	PC2			
**FVC**					
Model 0	2.17 (−1.98; 6.33)	−5.13 (−9.36; −0.91)	—	—	—
Model 1	—	—	1.78%	4.48	Reference
Model 5^a^	3.66 (−3.01; 10.3)	−1.33 (−7.87; 5.02)	0.04%	0.08	98.2%
**FEV**_**1**_					
Model 0	2.78 (−1.07; 6.63)	−3.11 (−7.02; 0.81)	—	—	—
Model 1	—	—	2.13%	4.53	Reference
Model 5^a^	1.54 (−4.58; 7.65)	1.07 (−4.93; 7.07)	0.40%	0.16	96.5%
**FEF**_**25%–75%**_					
Model 0	5.05 (−1.27; 11.4)	−0.50 (−6.94; 5.93)	—	—	—
Model 1	—	—	0.37%	2.14	Reference
Model 5^a^	−5.19 (−16.2; 5.77)	4.86 (−5.89; 15.6)	7.10E-7%	3.71E-6	>99.9%
**EBC pH**					
Model 0	0.02 (−0.20; 0.24)	−0.05 (−0.17; 0.17)	—	—	—
Model 1	—	—	2.04%	4.33	Reference
Model 5^a^	0.12 (−0.53; 0.77)	−0.002 (−0.65; 0.64)	9.32%	0.09	97.8%
**Exhaled NO**					
Model 0	0.20 (−0.02; 0.41)	−0.14 (−0.35; 0.07)	—	—	—
Model 1	—	—	3.98%	0.03	Reference
Model 5^b^	−0.12 (−0.68; 0.44)	−0.37 (−0.93; 0.18)	6.76%	0.05	−54.6%
**Baseline pupil diameter**					
Model 0	−0.09 (−0.32; 0.14)	−0.03 (−0.26; 0.19)	—	—	—
Model 1	—	—	20.3%	0.158	Reference
Model 5^c^	−0.12 (−0.84; 0.60)	0.02 (−0.70; 0.75)	22.1%	0.178	−12.5%
**Final pupil diameter**					
Model 0	−0.08 (−0.24; 0.08)	−0.10 (−0.26; 0.06)	—	—	—
Model 1	—	—	14.1%	0.053	Reference
Model 5^c^	−0.10 (−0.51; 0.31)	−0.07 (−0.48; 0.35)	15.1%	0.059	−10.3%
**ACV**					
Model 0	−0.11 (−0.29; 0.07)	0.17 (−0.01; 0.35)	—	—	—
Model 1	—	—	15.7%	0.075	Reference
Model 5^c^	−0.05 (−0.56; 0.45)	0.23 (−0.28; 0.74)	16.7%	0.081	−8.24%
**MCV**					
Model 0	−0.06 (−0.32; 0.19)	0.26 (0.01; 0.51)	—	—	—
Model 1	—	—	12.1%	0.122	Reference
Model 5^c^	0.03 (−0.61; 0.68)	0.26 (−0.39; 0.91)	12.7%	0.118	−6.12%
**Constriction amplitude**					
Model 0	−0.25 (−1.56; 1.04)	1.17 (−0.12; 2.45)	—	—	—
Model 1	—	—	8.38%	2.07	Reference
Model 5^c^	−0.07 (−2.80; 2.65)	1.50 (−1.26; 4.27)	7.52%	1.85	10.9%
**ADV**					
Model 0	−0.02 (−0.10; 0.07)	0.06 (−0.03; 0.14)	—	—	—
Model 1	—	—	8.57E-8%	8.10E-11	Reference
Model 5^c^	−0.02 (−0.13; 0.09)	0.06 (−0.05; 0.17)	7.82E-8%	7.48E-11	7.60%
**T75**					
Model 0	0.21 (0.01; 0.42)	−0.13 (−0.33; 0.06)	—	—	—
Model 1	—	—	4.53%	0.023	Reference
Model 5^c^	0.31 (−5.07E-4; 0.63)	−0.10 (−0.42; 0.22)	3.89%	0.020	13.0%

*corresponds to the proportion of between-schools variance that could be explained by exposure and individual characteristics; PC1: discontinuous dense urban fabric, discontinuous medium density urban land, green urban areas, and water bodies; PC2: construction sites, land without current use, and railways; 95% CI: 95% confidence interval; ICC: intra-class correlation coefficient; FVC: forced vital capacity; FEV_1_: forced expiratory volume in the first second of FVC; FEF_25–75_: forced expiratory flow in the middle portion of FVC; EBC: Exhaled breath condensate; ACV: Average constriction velocity; MCV: Maximum constriction velocity; ADV: Average dilation velocity; T75: the total time taken by the pupil to recover 75% of its initial resting diameter after it reached the peak of constriction.

Model 0 only included the PC1 and PC2 score; ^a^Model 1 is null model, baseline model without any exposure variable; Model 5^a^ is additionally adjusted for age, sex, asthma, WHO z-score for BMI and family history of asthma or allergy; Model 5^b^ is additionally adjusted for age, sex, asthma, atopy, WHO z-score for BMI and family history of asthma or allergy; Model 5^c^ is additionally adjusted for age, sex, asthma, and WHO z-score for BMI.

No significant associations were observed between green or built areas and pupillometry parameters. Still, a positive trend was found between built areas and pupillometry parasympathetic parameters (ACV, MCV and constriction amplitude; Supplementary Fig. S4a). After adjustment, estimates of ICCs for pupillometry suggested that between 0% and 22% of the total variance was at the school level. The neighbouring environment explained 6% of the effect of school on MCV, 8% of its effect on ADV, 11% of its effect on constriction amplitude and 13% of its effect on T75 (model 5, Table 1; Supplementary Fig. S4b).

Neighbourhood walkability explained >99.9% of the school effect on FVC, FEV_1_ and FEF_25–75%_. Regarding autonomic nervous system response, neighbourhood walkability explained 11% and 18% of the parasympathetic outcomes (constriction amplitude and MCV, respectively) and 7% and 29% of the pupillometry sympathetic parameters (ADV and T75, respectively) (Table 2; Supplementary Table S2). Lung function and exhaled NO decreased nonsignificantly with neighbourhood walkability (Supplementary Figs S5 and S7), while a positive association was observed for exhaled breath condensate pH level (Fig. S6). After adjustment for age, sex, asthma and WHO z-score for BMI, a significant negative association between walkability around schools and constriction amplitude (β = −1.62, 95% CI –2.87, −0.37) and T75 (β = −0.19, 95% CI –0.36, −0.02) was observed. Additionally, walkability showed a tendency to be associated with lower values of ACV, MCV and baseline pupil diameter (Supplementary Fig. S8a,b).

**Table 2:** Multilevel model analysis of the association between individual and walkability and lung function, pH, exhaled NO and pupillometry parameters explained by school.

Outcome	Walkabilityβ (95% CI)	School		
		ICC	Variance	Explained variation*
**FVC**				
Model 0	−0.58 (−2.79; 1.63)	—	—	—
Model 1	—	1.78%	4.48	Reference
Model 5^a^	−2.62 (−6.00; 0.77)	1.32E-6%	2.51E-6	>99.9%
**FEV**_**1**_				
Model 0	−1.02 (−3.07; 1.02)	—	—	—
Model 1	—	2.09%	4.53	Reference
Model 5^a^	−2.63 (−5.71; 0.46)	4.47E-7%	7.09E-7	>99.9%
**FEF**_**25%-75%**_				
Model 0	−1.27 (−4.74; 1.98)	—	—	—
Model 1	—	0.37%	2.14	Reference
Model 5^a^	−0.72 (−6.33; 4.89)	5.27E-7	2.77E-6	>99.9%
**EBC pH**				
Model 0	0.09 (−0.03; 0.21)	—	—	—
Model 1	—	3.48%	0.03	Reference
Model 5^a^	−0.004 (−0.34; 0.33)	8.52%	0.08	>−99.9%
**Exhaled NO**				
Model 0	−0.05 (−0.17; 0.07)	—	—	—
Model 1	—	3.98%	0.03	Reference
Model 5^b^	−0.07 (−0.38; 0.23)	8.07%	0.06	−90.6%
**Baseline pupil diameter**				
Model 0	−0.22 (−0.34; −0.09)	—	—	—
Model 1	—	20.4%	0.158	Reference
Model 5^c^	−0.18 (−0.54; 0.17)	22.1%	0.178	−12.5%
**Final pupil diameter**				
Model 0	−0.04 (−0.13; 0.05)	—	—	—
Model 1	—	14.1%	0.053	Reference
Model 5^c^	−0.05 (−0.26; 0.16)	15.1%	0.059	−10.3%
**ACV**				
Model 0	−0.22 (−0.32; −0.12)	—	—	—
Model 1	—	14.7%	0.070	Reference
Model 5^c^	−0.21 (−0.46; 0.03)	16.7%	0.081	−16.4%
**MCV**				
Model 0	−0.31 (−0.45; −0.17)	—	—	—
Model 1	—	12.3%	0.112	Reference
Model 5^c^	−0.29 (−0.60; 0.02)	10.1%	0.091	18.0%
**Constriction amplitude**				
Model 0	−1.94 (−2.65; −1.23)	—	—	—
Model 1	—	8.38%	2.073	Reference
Model 5^c^	−1.62 (−2.87; −0.37)	7.52%	1.847	10.9%
**ADV**				
Model 0	0.01 (−0.04; 0.06)	—	—	—
Model 1	—	8.57E-8%	8.10E-11	Reference
Model 5^c^	0.005 (−0.06; 0.07)	7.82E-8%	7.48E-11	7.60%
**T75**				
Model 0	−0.17 (−0.29; −0.06)	—	—	—
Model 1	—	4.53%	0.023	Reference
Model 5^c^	−0.19 (−0.36; −0.02)	3.19%	0.016	29.3%

*corresponds to the proportion of between-schools variance that could be explained by exposure and individual characteristics; 95% CI: 95% confidence interval; ICC: intra-class correlation coefficient; FVC: forced vital capacity; FEV_1_: forced expiratory volume in the first second of FVC; FEF_25–75_: forced expiratory flow in the middle portion of FVC; EBC: Exhaled breath condensate; ACV: Average constriction velocity; MCV: Maximum constriction velocity; ADV: Average dilation velocity; T75: the total time taken by the pupil to recover 75% of its initial resting diameter after it reached the peak of constriction.

Model 0 only included the PC1 and PC2 score; ^a^ Model 1 is null model, baseline model without any exposure variable; Model 5^a^ is additionally adjusted for age, sex, asthma, WHO z-score for BMI and family history of asthma or allergy; Model 5^a^ is additionally adjusted for age, sex, asthma, atopy, WHO z-score for BMI and family history of asthma or allergy; Model 5^c^ is additionally adjusted for age, sex, asthma, and WHO z-score for BMI.

## Discussion

We report for the first time an association among school neighbourhood environments, lung function, and autonomic function in children. Built areas around schools were inversely associated with children’s lung function, specifically forced vital capacity, in both crude and adjusted mixed-effect models. Moreover, a non-significant relationship between schools surrounding greenness and lung function parameters was observed. On the basis of our results, it is plausible that effects of environment on lung function may be partly neurogenically mediated, as schools’ neighbourhood walkability explained up to 14% and 30% of the effect of school on parasympathetic and sympathetic activity, respectively.

Our study has a few limitations. The cross-sectional design does not allow the establishment of causal relations or the analysis of cumulative exposure to different neighbourhoods. Furthermore, no on-site monitoring data regarding air pollution levels were measured, and we did not address the quality of green spaces, vegetation types or biodiversity. Nevertheless, several studies on urban environmental effects reported that land use could be used as an indicator of urban-related air pollution, such as traffic, without outdoor air monitoring^22,23^. Additionally, the use of an exposure metric based on urban land use thereby incorporates traffic-related emissions, but also includes other urban factors^22,23^. Rosenlund, *et al*.^24^ also found a reasonable agreement between land-use and traffic emissions. Nevertheless, neighbourhood land use patterns and walkability around schools were quantified numerically, avoiding bias related to participants’ perception of their neighbourhoods. Walkability is an objective measure of built environments and represents how friendly a neighbourhood area is to walking and bicycling; this measure is shaped by different urban-design features such as residential density, pedestrian-friendly design, street connectivity and diversity of neighbourhood land use^25^. Living in neighbourhoods characterized by higher walkability was found to be associated with more walking and cycling for transport and leisure and with moderate to vigorous physical activity^26^ and reduced obesity and overweight^27^. Walkable urban areas may offer health benefits, but may also come with health costs when exposure to air pollution is considered^28^. Our study considers only the walkability around schools, however, several studies have reported the impact of walkability around schools in planning school neighbourhoods (accessible schools with low traffic, sidewalks), in decisions that support the active commuting to school^29^, and also in the decrease of automobile dependence in childhood that carries over into adolescence and adulthood^29^. In addition, assessing the walkability around individual’s home may not necessarily reflect the facilities that they use or environments in which they are active^30^. Moreover, indicators of asthma severity, such as number of asthma attacks, attendance in emergency service and hospitalization due to asthma in the last 12 months, and asthma medication use were not considered. However, time-dependent exposure to the effect of school neighbourhoods is expected to be associated with severe exacerbation of asthma in asthmatic children. Nevertheless, it will be important to assess the effect of long-term exposure to school neighbourhoods to understand the extent of health effects. The potential selection bias is also a limitation; however, no significant differences were found between the children not included in the study and those included, being expected that our associations were most likely not biased. Additionally, we measured the effect of schools’ neighbourhoods using a robust statistical tool that allowed a multilevel approach, considering the complex relationship among the different levels of variables. Our results are also limited by low intraclass correlation coefficients (ICCs) to estimate the percent of total variance in outcomes between neighbourhoods generated by the variables of the multilevel analysis. However, even low ICCs may coexist with important fixed effects of contextual variables. Public health is full of examples of risk factors that explain very little inter-individual variance but are considered important predictors of health outcomes. Thus, as Duncan and colleagues^31^ have stated, even variables with low ICCs are considered important predictors of health outcomes and are compatible with important policy effects of neighbourhood characteristics on health. Since ICCs represent the proportion of the variance at the school level rather than individual, they may indicate to what extent school interventions and policies influence outcome-relevant individual predictors^32^. Our results suggest that the school neighbourhood explains an important portion of the variance for all outcomes suggesting that school-level changes may have an important impact on children health outcomes. Furthermore, higher ICCs suggest that the effect on lung function and autonomic nervous system activity in children may be predicted by school neighbourhood as well as characteristics of the children.

**Figure 1:**
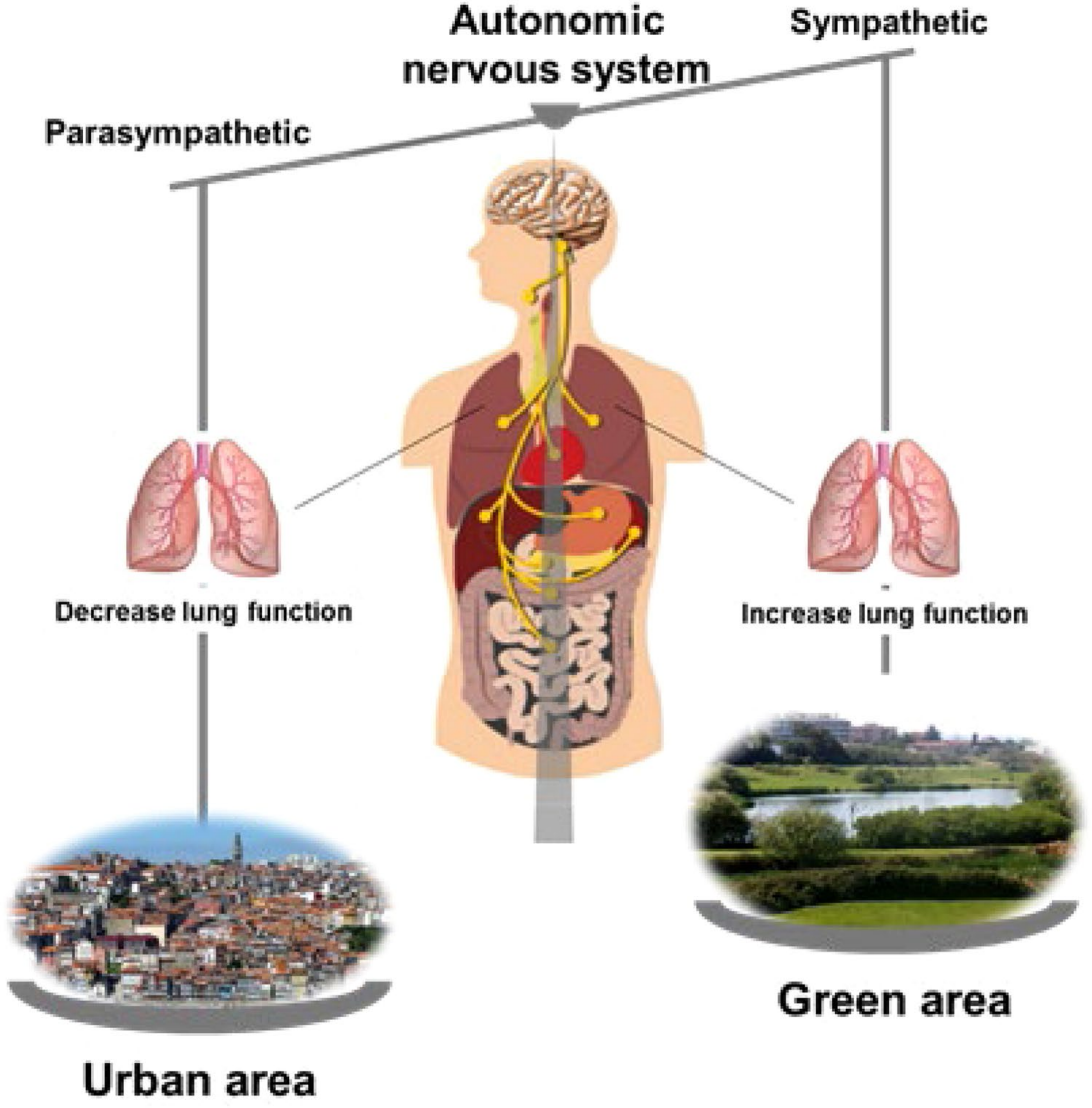
Environment-lung function interaction: a hypothesis focused on autonomic nervous system activity.

Our study has also important strengths. To our knowledge, this is the first community-based study evaluating the effect of schools’ neighbourhoods on lung function, airway reversibility and inflammation, and autonomic nervous system activity. Additionally, we performed a comprehensive clinical assessment with a large number of participants, including an assessment of autonomic status that allowed us to assess the children’s ability to respond to stress. Different studies have demonstrated that subjects with increased bronchial hyperresponsiveness have higher vagal tone, proposing that increased parasympathetic activity could predispose individuals to increased bronchomotor tone^33,34^. However, according to the European Respiratory Society (ERS) and American Thoracic Society (ATS) guidelines, bronchial responsiveness tests are suitable for adults and older children. Young children have a short concentration span and relatively poor cooperation on these pulmonary function tests^35^. Although this study assessed the effect of schools’ neighbourhoods on lung function, asthma is characterized by airflow obstruction^36^ with changes in lung function parameters^37^. Regular assessment of lung function, namely FEV1, might help to identify children at risk for developing a progressive decline in airflow^38^. Furthermore, airway obstruction in children is often triggered by environmental factors. Previous studies have reported associations between exposure to urban areas and adverse respiratory health effects, especially in children, with the ESCAPE meta-analysis of data for 5921 children from five European birth cohorts reporting that annual exposures to NO_2_, NO_x_, PM_10_, and PM_2.5_ were associated with reduced lung function^2^. The negative impact of exposure to urban environment has also been further reinforced by Mudway, *et al*.^3^, in which exposure to urban air, particularly to NO_x_ and NO_2_, was inversely associated with lung function, and by Gauderman, *et al*.^39^, which showed that reductions in pollution delivered significant improvements in FEV1 and FVC. Taken together, our findings may contribute support for plans of action aiming to improve urbanization plans in cities and thereby improve respiratory health in children. This study assessed the effects of green and built areas within an urban context, while most previous studies of environmental impacts on asthma and allergies have reported differences between urban and rural environments. Our results suggested that the presence of urban green areas has a positive effect on lung function. Our findings suggest that autonomic nervous system may play a role in mediating the interaction between the environment and the individual (Fig. 1).

Several studies have addressed the use of pupillometry to measure autonomic nervous system activity, using different indices from the constriction (parasympathetic) and dilation (sympathetic) phases in response to light^40,41^. Autonomic balance can change with an increase in vagal activity by the simple act of viewing natural scenes, as has been recently shown by Gladwell and colleagues^42^. In their study, a slideshow containing natural scenes, compared with another that incorporated built or urban scenes lacking green space, induced changes in autonomic control via increases in vagal modulation^42^. Additionally, a review of field experiments conducted in 24 forests across Japan on the effects of shinrin-yoku (taking in the forest atmosphere, or “forest bathing”) showed that forest environments could lower concentrations of cortisol, decrease heart rate and blood pressure, increase parasympathetic nerve activity, and lower sympathetic activity compared with city settings^43^. However, comparisons of our findings with those of other studies are limited by the different methodologies used to assess autonomic nervous system activity and environmental exposure. The differences found in autonomic nervous system response may be related to the effects of the type of natural settings (parks, gardens, sports fields, forests, tree corridors, or other green space types) and the time spent in each area^44^. In this study, we assess the effect of green areas in an urban environment, where green areas may be smaller and where children are expected to spend less time, as opposed to previous studies in Japan that reported the effect of green areas outside the city, specifically, in forest areas^43^. Thus, urban green areas may have a different effect on autonomic nervous system activity. In addition, several animal studies also highlight the role of autonomic nervous system balance in the interaction between the environment and the individual^45,46^.

In our study, built areas around schools adversely affect lung function but not eosinophilic airway inflammation. While the effect of outdoor air pollution on asthma and related symptoms is already recognized, the underlying mechanisms remain unclear^47^. Air pollutants, such as particulate matter, ozone, and nitrogen dioxide, can activate the transient receptor potential (TRP) cation channels on airway C-fibre sensory nerves, namely, TRP vanilloid type 1 (TRPV1) and ankyrin (TRPA1), and cause several responses, such as bronchoconstriction, mucus secretion, airway irritation, and cough, mediated by the efferent pathways of the autonomic nervous system^15^. Akopian *et al*. and Geppetti *et al*. described the association between environmental pollutants and the expression of TRP channels in pulmonary disease, providing evidence for the role of autonomic nervous system activity in the regulation of airway function^15,48^. Therefore, exposure to air pollution is expected to be higher in built areas around schools’ neighbourhood than in green areas and may be associated with an activation and/or increased expression of TRPV1 and TRPA1. This may, in turn, result in increased parasympathetic activity with subsequent decreased lung function.

Recent studies have shown evidence of beneficial associations between greenness and health outcomes. Urban green spaces not only provide balance for ecosystems but also promote physical activity, psychological well-being, and public health in urban populations^49^. Greenness may influence health by promoting physical activity and opportunities for social interactions, decreasing the risk of many chronic diseases and psychophysiological stress and reducing air pollution levels, noise, and heat exposure^50^. In children, exposure to green areas has been associated with reduced obesity and sedentary behaviours^50,51^. Ruokolainen and colleagues have shown the amount of forest and agricultural land around homes to be inversely associated with the risk of atopy in children^52^. These findings provide support for a role of natural environmental on the regulation of the T_H_1, T_H_2 immune response mediated by the children commensal microbiota^52,53^. Furthermore, in children living in greener areas of Vancouver, as measured by the normalized differential vegetation index, had a slightly reduced risk of incidence of asthma (aOR = 0.96; 95% CI 0.93–0.99)^54^. Similarly, lower asthma prevalence in areas with greater tree density in New York City has been reported^55^. Nevertheless, no individual-level studies are available to compare with our findings; however, these associations are similar to the reported results of previous studies on the association between greenness and asthma. Although several studies reported the role of greenness as a buffer against exposure to air pollution and the positive effect of greenspaces in urban context^50,56^, air pollution can also affect plant health and functions and limit pollutant dispersion and thus increase local pollutant concentration^57,58^.

Exploring the effects of schools’ neighbourhoods is crucial for planning, defining guidelines, and making recommendations to cities planners and decision makers in order to create healthier and sustainable urban environments, with potential to protect citizens against the development of asthma and allergic diseases. Thus, our results meet the goals of the WHO European Healthy Cities Network, demonstrating the importance of policies and scientific evidence for health development, public health and urban regeneration to promote and protect human health. Furthermore, this study may contribute to changes in urban environments, such as introducing or improving existing green spaces (parks, green corridors, urban gardens or green exercise programmes), which would provide opportunities for health improvement and social interactions, thus adding to the additional benefits of green urban areas to the local economies, sustainability and self-sufficiency of cities.

The present study demonstrates that the neighbourhoods around schools may have an effect on child health, specifically on lung function and on autonomic nervous system activity. The effects on lung function may be potentially mediated by an increase in parasympathetic activity. These results also underline the positive health effects of green areas in school neighbourhoods, contributing to the implementation of urban planning policies and practices that may promote a healthy lifestyle and reconnection with nature.

## Methods

The present study included participants from a cross-sectional study assembled in Porto, Portugal. The 20 schools with the highest number of students were selected from a total of 53 primary schools, corresponding to a total of 71 assessed classrooms (see the methods section in the Online Repository). The evaluations included a questionnaire and a physical and clinical assessment of children. The University Health Ethics Committee approved the study, and informed consent was obtained from the children’s legal guardians. All research was performed in accordance with the Declaration of Helsinki.

### Questionnaire

The evaluation included a self-administered ISAAC-based questionnaire filled out by parents, covering information on social, demographic and behavioural characteristics and questions regarding the respiratory/allergic health of the children (ever had and over the past 12 months) (see the methods section in the Online Repository).

### Physical and clinical assessment

A physical and clinical assessment was also performed at each primary school by a trained health professional. Spirometry with bronchodilation, exhaled level of nitric oxide, exhaled breath condensate (EBC), pupillometry, skin prick test (SPT), weight, and height were measured for all participants (physical and clinical assessment methods are detailed in the Supplementary Material).

Pupillary measurements were taken with a portable infrared PLR-200 pupillometer (NeurOptics PLR-200™ Pupillometer, NeurOptics Inc., CA). Children spent at least 15 min in a semi-dark and quiet room to allow pupillary adjustment to the low level of light, after which they were instructed to focus with the eye that was not being tested on a small object three metres away, keeping their head straight and eyes wide open during targeting and measurement. Light-emitting diodes briefly illuminated the eye once with a peak wavelength of 180 nm. One pupil light response curve for each eye was recorded for each child. Data on the diameter (millimetres) of the pupil before the light stimulus (initial) and at constriction peak (minimal), relative constriction amplitude (%), maximum constriction velocity (MCV), average constriction (ACV) and dilation (ADV) velocities (mm/s), and total time (seconds) taken by the pupil to recover 75% of its initial resting diameter after it reached the peak of constriction (T75) were recorded for each child. Pupillometry is a simple, noninvasive technique that can provide valuable data concerning the functioning of both branches of the autonomic nervous system. Pupil diameter, ACV, MCV, and constriction amplitude are related to parasympathetic activity, while ADV and T75 are measures of sympathetic activity^59^ (Supplementary Material).

### Urban land use

**Figure 2:**
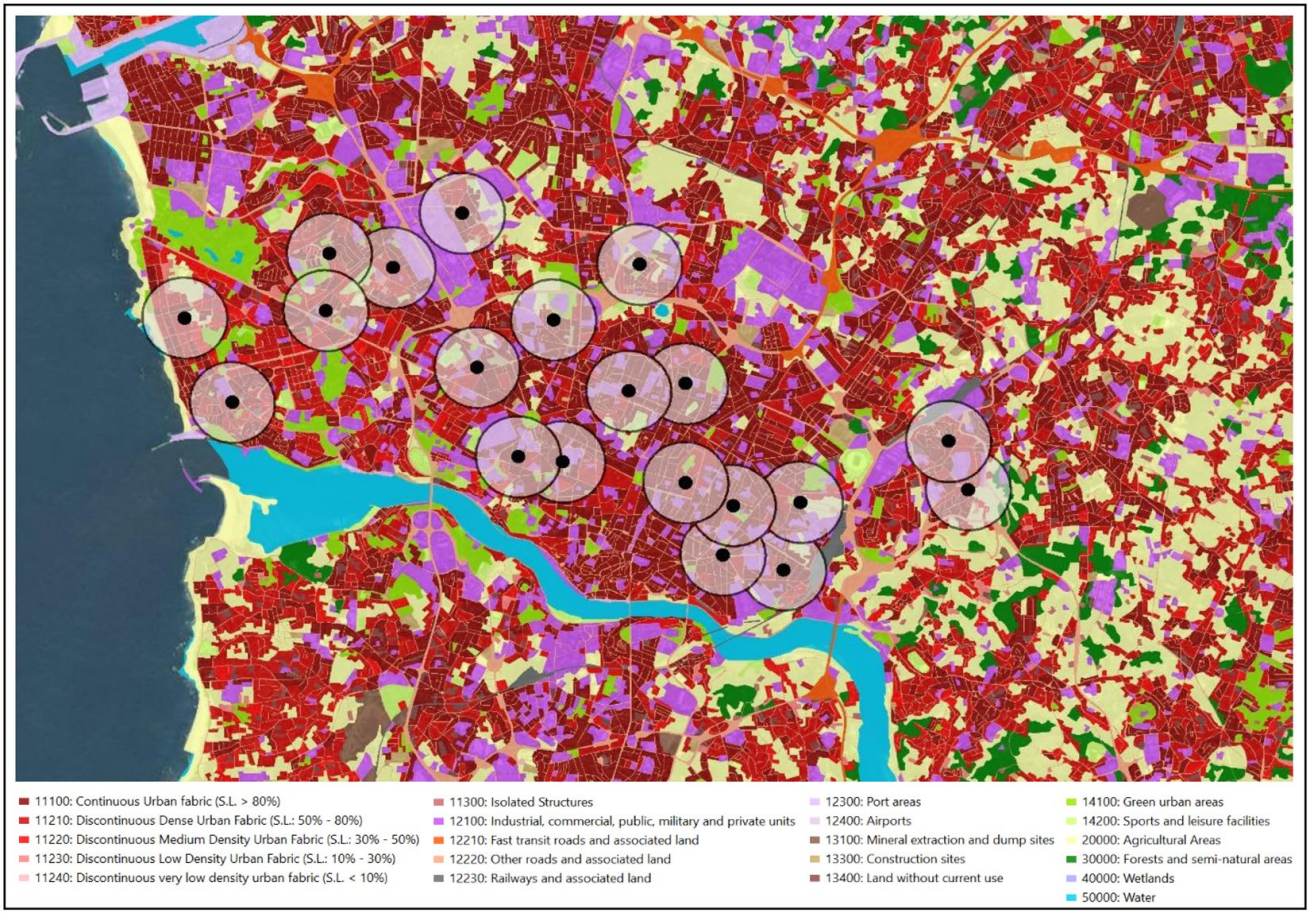
Neighbourhood land use around the 20 evaluated primary schools in Porto. Each school was represented by a point and a circular buffer of 500 metres. For this assessment, we used the ArcGIS 10.4 Network Analyst tool (Environmental Systems Research Institute, ESRI, Redlands, CA, USA).

The land use near each school was calculated on the basis of the European Urban Atlas using a geographical information system (GIS). The Urban Atlas (https://www.eea.europa.eu/data-and-maps/data/copernicus-land-monitoring-service-urban-atlas) city information is currently the most up-to-date, harmonized database for the European Union countries, offering a high-resolution land-use map of cities (population ≥ 100,000), mapped using a total of 20 land use classes (Supplementary Table S3)^60^. A circular buffer of 500 metres around each participant’s primary school address was created (Fig. 2). This buffer was based on reasonable walking distances described by Browson and colleagues^61^, corresponding to approximately 6 minutes’ walking distance for children.

### Walkability

The term walkability has been used to conceptualise a combination of built environment factors such as street connectivity, residential density, net area retail and land use mix, that are conducive to walking (i.e. walking to destinations, including work, school, shopping)^25^. Walkability is an indicator of how user-friendly a neighbourhood area is for walking and biking^25^.

The walkability index was calculated on the basis street connectivity, residential density, and land use mix (expressed as an index of entropy), within the 500-metre buffer. This calculation has been previously described and determined across Porto neighbourhoods by Ribeiro and colleagues^62^. Briefly, the street connectivity was calculated from the density of street junctions within the primary school’s neighbourhood. Residential density in each neighbourhood was obtained by calculating the density (number/area) of households. Land use mix expresses the diversity of land-use types in each neighbourhood (commercial, residential, recreational/leisure, business/industrial, educational and others).

After these three components were calculated for each neighbourhood (connectivity, residential density, and land use mix), the raw values were normalized using z-scores. The walkability index was calculated according to the following formula:

$${\rm{Walkability}}=(2\ast {\rm{z}}\mbox{--}{\rm{connectivity}})+({\rm{z}}\mbox{--}{\rm{residential}}\,{\rm{density}})+({\rm{z}}\mbox{--}{\rm{land}}\,{\rm{use}}\,{\rm{mix}})$$

**Figure 3:**
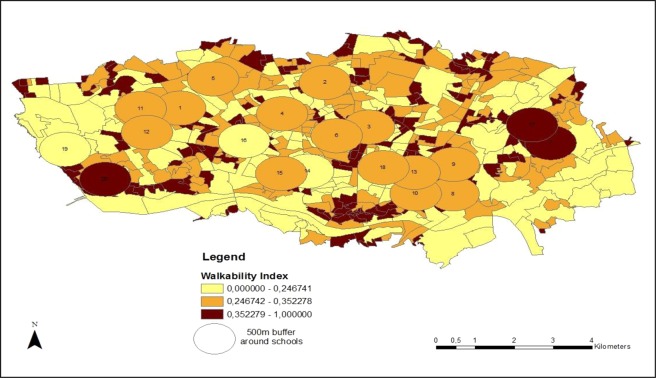
Neighbourhood walkability index around each primary school. Each school, represented by a number and a circular buffer of 500 metres, was characterized according to tertiles of neighbourhood walkability. For this assessment, we used the ArcGIS 10.4 Network Analyst tool (Environmental Systems Research Institute, ESRI, Redlands, CA, USA).

This formula is an adapted version of the formula of Frank and colleagues^25^. Next, the values were normalized between zero (least walkable) and one (most walkable). Primary schools’ neighbourhoods were characterized according to tertiles (from low to high) of neighbourhood walkability (Fig. 3).

### Participants

In total, 1602 children (7–12 years old), all in the 3^rd^ and/or 4^th^ grades, were invited to participate. Among them, 686 did not return the signed informed consent form and 58 refused to undergo clinical tests. Among the remaining 858 children, 146 were excluded owing to poor-quality data. Thus, this study was based on data from 701 children (50.9% girls). Of those, almost 9.4% reported wheezing symptoms, and 12% reported cough symptoms. The prevalence of asthma, rhinitis, current rhinitis, and atopy were 10.7%, 13.0%, 30.4%, and 35.5%, respectively (Table 3).

**Table 3:** Characteristics of the participants.

Characteristics	Total n = 701	Girls n = 357	Boys n = 344	*p* value*
Age [years (mean ± SD)]	9 ± 0.8	9 ± 0.8	9 ± 0.8	0.574
Wheezing symptoms [n (%)]	66 (9.4)	34 (9.5)	32 (9.3)	0.920
Cough symptoms [n (%)]	82 (11.7)	45 (12.6)	37 (10.8)	0.446
Asthma [n (%)]**	75 (10.7)	47 (13.2)	28 (8.1)	0.037
Rhinitis [n (%)]	81 (13.0)	36 (11.3)	45 (14.8)	0.233
Current rhinitis [n (%)]	69 (30.4)	32 (26.8)	37 (34.3)	0.611
Atopy [n (%)]	245 (355)	128 (36.4)	117 (34.5)	0.580
BMI [n (%)]				0.978
Underweight	33 (4.7)	17 (4.8)	16 (4.7)	
Normal weight	478 (68.2)	242 (67.8)	236 (68.6)	
Overweight	108 (15.4)	57 (16.0)	51 (14.8)	
Obese	82 (11.7)	41 (11.5)	41 (11.9)	
EBC pH	6.9 (0.9)	6.8 (0.9)	6.9 (0.9)	0.105
Exhaled NO (ppb)	17.1 (20.1)	14.6 (15.5)	19.6 (23.7)	0.010
Lung function				
FVC (%)	106.8 (15.7)	113.4 (14.9)	99.7 (13.2)	< 0.0001
FEV_1_ (%)	102.8 (14.6)	109.3 (13.8)	95.8 (11.8)	< 0.0001
FEF_25–75_ (%)	98.1 (24.3)	98.6 (24.3)	97.6 (24.2)	0.486
Pupillometry				
Baseline pupil diameter (mm)	5.3 (0.9)	5.2 (0.9)	5.3 (0.9)	0.137
Final pupil diameter (mm)	3.4 (0.6)	3.4 (0.6)	3.4 (0.6)	0.745
ACV (mm/s)	−3.9 0.7)	−3.9 (0.7)	−4.0 (0.7)	0.096
MCV (mm/s)	−5.2 (1.0)	−5.1 (1.0)	−5.4 (1.0)	0.021
Constriction amplitude (%)	35.1 (4.8)	34.5 (4.8)	35.6 (4.7)	0.017
ADV (mm/s)	1.1 (0.3)	1.2 (0.3)	1.1 (0.3)	0.750
T75 (s)	1.7 (0.7)	1.7 (0.7)	1.8 (0.7)	0.203

Data reported as median (interquartile range) unless otherwise stated. BMI: body mass index; FVC: forced vital capacity; FEV_1_: forced expiratory volume in the first second of FVC; FEF_25–75_: forced expiratory flow in the middle portion of FVC; EBC: Exhaled breath condensate; ACV: Average constriction velocity; MCV: Maximum constriction velocity; ADV: Average dilation velocity; T75: the total time taken by the pupil to recover 75 of its initial resting diameter after it reached the peak of constriction.

### Data analysis

The Kolmogorov-Smirnov test was used to check continuous variables for normality. The Mann-Whitney test was used to compare variables between girls and boys. Significant differences were defined according to an α-value of 5% (p < 0.05).

Principal component analysis (PCA) was used to identify major neighbourhood patterns based on 20 land use classes. Varimax rotation was performed to simplify the interpretation of the factor loading structure. A fixed number of factors were extracted, and two principal components were selected on that basis. The PCA divided neighbourhood land use around schools into two principal components (PC1, PC2) (Supplementary Table S4). Between the two factors, PC1 had higher absolute correlation with discontinuous dense urban fabric, discontinuous medium-density urban land, green urban areas, and water bodies while PC2 had higher absolute correlation with construction sites, land without current use, and railways. PC1 was characterized as green urban areas, and PC2 as built areas. Afterwards, the PC1 and PC2 scores were ranked from 1 to 20, and the rank numbers were divided by 20.

Mixed-effect models with a random effect of school were used to measure the effect of schools on lung function, airway inflammation and autonomic nervous system in children. The intraclass correlation coefficient (ICC) and the proportion of explained variation were used to quantify the effect of schools and to quantify the effect of individual, neighbouring environment, and walkability on the school effect. The effect of schools’ neighbourhoods on children health were analysed using a multilevel with individual-level and neighbourhood-level factors. All individual- and neighbourhood-level (as median values) factors were used in the multilevel analysis as independent variables. Five models were considered for the analysis: crude model (model 0 and 1), neighbourhood-level model (model 2), and an individual-neighbourhood level model (mixed effects model, models 3–5). Model 0, only included the PC1 and PC2 score or walkability; Model 1, the null model, baseline model without any exposure variable; Model 2 is adjusted for PC 1 and PC2 score or walkability; Model 3 is additionally adjusted for age, sex and asthma and atopy for exhaled NO; Model 4 is additionally adjusted for WHO z-score for BMI; and Model 5 is additionally adjusted for parental education level and family history of asthma or allergy for lung function parameters, exhaled NO and EBC pH. To minimize errors due to multiple comparisons, the Bonferroni correction was used to assess statistical significance. PCA, mixed-effect models and ICC were computed using the software RStudio, version 1.0.

## Supplementary information

Supplementary material
