# Supplementary material for: School environment associates with lung function and autonomic nervous system activity in children: a cross-sectional study
Source: Sci Rep. 2019 Oct 22;9:15156. doi: 10.1038/s41598-019-51659-y (PMC6805928; doi:10.1038/s41598-019-51659-y)
Supplement: Supplementary file 1 — Supplementary material [file 41598_2019_51659_MOESM1_ESM.pdf]

Online supplement

School environment associates with lung function and autonomic nervous system activity in children: a cross-sectional study

Inês Paciência[1,2,3], João Cavaleiro Rufo[1,2,3], Diana Silva[1], Carla Martins[1], Francisca Mendes[1], Tiago Rama[1], Ana Rodolfo[1], Joana Madureira[2], Luís Delgado[1], Eduardo de Oliveira Fernandes[2], Patrícia Padrão[3,4], Pedro Moreira[3,4], Milton Severo[3,5], Maria Fátima Pina[6,7,8], João Paulo Teixeira[3,9], Henrique Barros[3,5], Lasse Ruokolainen[10], Tari Haahtela[11], André Moreira[1,3,4]

Supplementary Materials

Supplementary methodology

Table S1: Multilevel model analysis of association between individual and neighboring environment and lung function, pH and pupillometry parameters explained by school.

Table S2: Multilevel model analysis of association between individual and walkability and lung function, pH and pupillometry parameters explained by school.

Table S3: Characterization of neighborhood land use around evaluated schools.

Table S4: Rotated Component Matrix.

Figure S1: Association between neighborhood around school and lung function parameters.

Figure S2: Association between neighborhood around school and EBC pH.

Figure S3: Association between neighborhood around school and exhaled level of nitric oxide.

Figure S4a: Association between neighborhood around school and autonomic nervous system activity.

Figure S4b: Association between neighborhood around school and autonomic nervous system activity.

Figure S5: Association between walkability index around school and lung function parameters.

Figure S6: Association between walkability index around school and EBC pH.

Figure S7: Association between walkability index around school and exhaled level of nitric oxide.

Figure S8: Association between walkability index around school and autonomic nervous system activity.

Figure S8b: Association between walkability index around school and autonomic nervous system activity.

Methods

From the 53 public primary schools of Porto Municipality, 20 schools with the highest number of students were selected. In each school, four classrooms of 3rd and/or 4th grades were selected among those with similar conditions and representative of the school building. Classrooms were selected based on ISAAC Phase II criteria, where children of this age group were more likely to understand the procedures than 6-7-year-old children and to be more compliant that 13-14 year old adolescents,[1] highest density of occupation, full-week occupation time by the same class, and, if possible, on the location of classrooms on different floors[2].

Questionnaire

Wheezing or cough symptoms were defined based on a positive answer to the questions “In the past 12 months, has your child had wheezing or whistling in the chest?” or to any of the two following questions “In the last 12 months, has your child suffered coughing at night?” or “In last year, has your child suffered coughing more than three months?”, respectively. Children were considered to have rhinitis if answered yes to the question “Has your child ever had a problem with sneezing, or a runny nose or blocked nose when he/she did not have a cold or the flu?” and a positive skin prick test (SPT). Current rhinitis was defined as a positive answer to the question “In the past 12 months, has your child had a problem with sneezing, or a runny nose or blocked nose when he/she did not have a cold or the flu?” and a positive SPT. Family history of asthma or allergy was recorded as a positive answer to the question “Are there any allergic disorders in the family, including asthma and allergies”.

Parental education level was used as socioeconomic status and was recorded as the number of successfully completed years of formal schooling, and children were classified according to the parent with the higher education level. Parental education level was categorized into three classes: ≤ 9 years; ≥ 10 years and ≤ 12 years; and > 12 years.

Physical and clinical assessment

Lung function and airway reversibility were assessed by spirometry according to ATS/ERS guidelines[3], using a portable spirometer (MIR Spirobank, A23-04003237) before and after 15 minutes of inhalation of 400µg of salbutamol. The predictive values for the spirometry parameters were calculated based on the GLI 2012 reference data[4] using GLI-2012 Excel Sheet Calculator[5]. Asthma was defined by at least a 12% increase in FEV1 after bronchodilation and over 200mL, or asthma diagnosed by a physician and reported symptoms (wheezing, dyspnoea or dry cough) in the past 12 months.

Airway inflammation was assessed measuring the fractional exhaled nitric oxide level using a NObreath analyzer (Bedfont Scienctific Ltd) in accordance with the ATS guidelines[6]. Exhaled breath

condensate (EBC) was also collected from the children by breathing (regular tidal volumes and respiratory rate) 10 to 15 minutes to an exhaled air condensing system (portable Turbo DECCS)[7]. EBC samples were stored at 4 ºC after collection, for up to 2 hours, and then transferred to sterile tubes and stored at -80ºC until laboratorial analysis. EBC samples were frozen for up to 2 years before analysis. We could not exclude the possibility that some biomarkers might start to degrade during storage, however it has been demonstrated that pH in the deaerated EBC samples is not affected by the duration of storage[8-11] and the length of time at room temperature before storage or analysis.[10] EBC pH was measured before and 10 minutes after deaeration with oxygen using a calibrated pH meter (pHenomenal® pH 1100 H) with an accuracy of ±0·005 pH. Deaeration was performed by bubbling oxygen through the sample for 10 minutes at 0·3 L/min[12,13]. The pH meter was calibrated before each measurement using solutions with pH values of 4, 7, and 10.

Skin-prick-tests were performed on children forearm using a QuickTestTM applicator containing house dust mite, mix of weeds, mix of grasses, cat dander, dog dander and *Alternaria alternata*, negative control, and a positive control consisting of histamine at 10mg/mL (Hall Allergy, Netherlands). Results were read 15 minutes afterwards and positive wheals are those exceeding 3 mm in diameter greater than the negative control. Atopy was defined by a positive SPT to at least one of the allergens[14].

Weight was measured using a digital scale (Tanita™ BC-418 Segmental Body Analyzer) and recorded in kilograms (Kg) and height in centimeters (cm) was measured using a portable stadiometer. Body mass index (BMI) was calculated using the ratio of weight/height^2 (Kg/m^2) and classified according to age- and sex-specific percentiles defined by the US Centres for Disease Control and Prevention (CDC)[15].

Pupil diameter, average and maximum construction velocity (ACV and MCV), and constriction amplitude are related to parasympathetic activity, while average dilation velocity (ADV) and the total time taken by the pupil to recover 75% of its initial resting diameter after it reached the peak of constriction (T75) are measures of sympathetic activity[16]. According to Purves D, Augustine GJ[17], the pupil light reflex is the reflex by which a change in pupil size occurs in response to light intensity. Under the direct control of the autonomic nervous system (ANS), the pupil light reflex reflects the balance between the parasympathetic (PNS) and sympathetic nervous system (SNS)[17]. The pupil light reflex pathway has been previously reported by Wang, Zekveld[18], showing that as response to light reflex, neurons of the PNS innervate circular fibers of the iris, causing pupillary constriction, whereas excitation by SNS neurons causes the radial fibers to produce dilation of the pupil. Therefore, the pupillary response to an external light stimulus might provide an indirect means to assess the ANS activity. Previous studies have proposed pupillometry as a simpler, noninvasive and sensitive tool the detect changes in ANS activity[16,19-21].

Outcome			School		
	β (95% CI)		ICC	Variance	Explained variation*
	PC1	PC2			
**FVC**					
**Model 0**	2·17 (-1·98; 6·33)	-5·13 (-9·36; -0·91)	---	---	---
**Model 1**[a]	---	---	1·78%	4·48	Reference
**Model 2**[b]	2·04 (-3·20; 7·29)	-4·98 (-10·3; 0·35)	1·11%	2·80	37·6%
**Model 3**[c]	2·12 (-2·17; 6·96)	-5·09 (-10·0; -0·15)	1·39%	2·78	38·0%
**Model 4**[e]	2·39 (-2·61; 7·38)	-4·62 (-9·73; 0·48)	1·75%	3·44	23·2%
**FEV_1**					
**Model 0**	2·78 (-1·07; 6·63)	-3·11 (-7·02; 0·81)	---	---	---
**Model 1**[a]	---	---	2·13%	4·53	Reference
**Model 2**[b]	2·56 (-2·77; 7·89)	-2·92 (-8·33; 2·49)	2·05%	4·16	8·17%
**Model 3**[c]	2·71 (-1·71; 7·13)	-2·91 (-7·42; 1·61)	1·58%	2·49	44·9%
**Model 4**[e]	2·91 (-1·65; 7·47)	-2·55 (-7·21; 2·11)	1·73%	3·00	33·6%
**$FEF_{25\%-75\%}$**					
**Model 0**	5·05 (-1·27; 11·4)	-0·50 (-6·94; 5·93)	---	---	---
**Model 1**[a]	---	---	0·37%	2·14	Reference
**Model 2**[b]	4·99 (-2·06; 12·0)	-0·48 (-7·65; 6·70)	0·22%	2·30	39·1%
**Model 3**[c]	5·08 (-1·53; 11·7)	0·37 (-6·42; 7·16)	2·73E-6%	1·50E-5	>99·9%
**Model 4**[e]	5·09 (-1·53; 11·7)	0·38 (-6·43; 7·19)	2·96E-6%	1·63E-5	>99·9%
**EBC pH**					
**Model 0**	0·02 (-0·20; 0·24)	-0·05 (-0·17; 0·17)	---	---	---
**Model 1**[a]	---	---	2·04%	4·33	Reference
**Model 2**[b]	0·06 (-0·34; 0·46)	-0·10 (-0·50; 0·30)	2·04%	4·33	0%
**Model 3**[c]	0·05 (-0·31; 0·41)	-0·08 (-0·44; 0·28)	2·88%	0·03	99·4%
**Model 4**[e]	0·05 (-0·31; 0·42)	-0·06 (-0·43; 0·30)	2·98%	0·03	99·4%
**Exhaled NO**					
**Model 0**	0·20 (-0·02; 0·41)	-0·14 (-0·35; 0·07)	---	---	---
**Model 1**[a]	---	---	3·98%	0·03	Reference
**Model 2**[b]	0·16 (-0·23; 0·54)	-0·10 (-0·49; 0·29)	4·21%	0·04	-6·16%
**Model 3**[d]	0·19 (-0·22; 0·59)	-0·16 (-0·56; 0·25)	4·81%	0·04	-16·8%
**Model 4**[e]	0·19 (-0·22; 0·59)	-0·14 (-0·55; 0·27)	4·97%	0·04	-20·7%
**Baseline pupil diameter**					
**Model 0**	-0·09 (-0·32; 0·14)	-0·03 (-0·26; 0·19)	---	---	---
**Model 1**[a]	---	---	20·3%	0·158	Reference
**Model 2**[b]	-0·11 (-0·84; 0·62)	0·01 (-0·72; 0·74)	22·3%	0·178	-12·5%
**Model 3**[c]	-0·10 (-0·84; 0·63)	0·06 (-0·68; 0·79)	22·1%	0·178	-12·6%
**Model 4**[e]	-0·10 (-0·84; 0·63)	0·06 (-0·67; 0·80)	22·1%	0·178	-12·5%
**Final pupil diameter**					
**Model 0**	-0·08 (-0·24; 0·08)	-0·10 (-0·26; 0·06)	---	---	---
**Model 1**[a]	---	---	14·1%	0·053	Reference
**Model 2**[b]	-0·09 (-0·52; 0·34)	-0·70 (-0·50; 0·37)	15·4%	0·059	-11·3%
**Model 3**[c]	-0·09 (-0·53; 0·34)	-0·05 (-0·59; 0·39)	15·1%	0·059	-10·3%
**Model 4**[e]	-0·09 (-0·53; 0·34)	-0·05 (-0·49; 0·39)	15·1%	0·059	-10·3%
**ACV**					
**Model 0**	-0·11 (-0·29; 0·07)	0·17 (-0·01; 0·35)	---	---	---
**Model 1**[a]	---	---	15·7%	0·075	Reference
**Model 2**[b]	-0·81 (-0·59; 0·42)	0·16 (-0·35; 0·67)	16·9%	0·082	-9·49%
**Model 3**[c]	-0·06 (-0·57; 0·45)	0·18 (-0·33; 0·69)	16·7%	0·081	-8·39%
**Model 4**[e]	-0·06 (-0·56; 0·45)	0·19 (-0·31; 0·70)	16·7%	0·081	-8·06%
**MCV**					
**Model 0**	-0·06 (-0·32; 0·19)	0·26 (0·01; 0·51)	---	---	---
**Model 1**[a]	---	---	12·1%	0·122	Reference
**Model 2**[b]	-0·01 (-0·64; 0·63)	0·21 (-0·42; 0·85)	13·2%	0·123	-10·2%
**Model 3**[c]	0·01 (-0·59; 0·61)	0·23 (-0·36; 0·84)	11·5%	0·106	5·27%
**Model 4**[e]	0·01 (-0·59: 0·60)	0·25 (-0·35; 0·85)	11·5%	0·106	5·94%
**Constriction amplitude**					
**Model 0**	-0·25 (-1·56; 1·04)	1·17 (-0·12; 2·45)	---	---	---
**Model 1**[a]	---	---	8·38%	2·07	Reference
**Model 2**[b]	-0·28 (-3·11; 2·55)	1·18 (-1·66; 4·02)	9·08%	2·26	-9·19%
**Model 3**[c]	-0·09 (-2·81; 2·63)	1·41 (-1·33; 4·14)	7·60%	1·87	9·79%
**Model 4**[e]	-0·09 (-2·80; 2·62)	1·48 (-1·25; 4·21)	7·52%	1·85	10·9%

**ADV**					
**Model 0**	-0·02 (-0·10; 0·07)	0·06 (-0·03; 0·14)	---	---	---
**Model 1[a]**	---	---	8·57E-8%	8·10E-11	Reference
**Model 2[b]**	-0·02 (-0·11; 0·08)	0·06 (-0·03; 0·15)	1·01E-7%	9·52E-11	-17·6%
**Model 3[c]**	-0·01 (-0·11; 0·09)	0·08 (-0·01; 0·18)	7·98E-8%	7·54E-11	6·81%
**Model 4[e]**	-0·01 (-0·11; 0·09)	0·07 (-0·03; 0·17)	7·92E-8%	7·48E-11	7·60%
**T75**					
**Model 0**	0·21 (0·01; 0·42)	-0·13 (-0·33; 0·06)	---	---	---
**Model 1[a]**	---	---	4·53%	0·023	Reference
**Model 2[b]**	0·20 (-0·13; 0·53)	-0·12 (-0·45; 0·21)	4·37%	0·022	3·62%
**Model 3[c]**	0·20 (-0·14; 0·55)	-0·12 (-0·47; 0·22)	3·98%	0·020	11·3%
**Model 4[e]**	0·20 (-0·14; 0·54)	-0·14 (-0·49; 0·20)	3·89%	0·020	13·0%

Table S1. Multilevel model analysis of association between individual and neighboring environment and lung function, pH, exhaled NO and pupillometry parameters explained by school.

*corresponds to the proportion of between-schools variance that could be explained by exposure and individual characteristics; PC1: discontinuous dense urban fabric, discontinuous medium density urban land, green urban areas, and water bodies; PC2: construction sites, land without current use, and railways; 95% CI: 95% confidence interval; ICC: intra-class correlation coefficient; FVC: forced vital capacity; FEV_1: forced expiratory volume in the first second of FVC; $FEF_{25\%-75\%}$: forced expiratory flow in the middle portion of FVC; EBC: Exhaled breath condensate; ACV: Average constriction velocity; MCV: Maximum constriction velocity; ADV: Average dilation velocity; T75: the total time taken by the pupil to recover 75% of its initial resting diameter after it reached the peak of constriction.

Model 0 only included the PC1 and PC2 score; [a] Model 1 is null model, baseline model without any exposure variable; [b] Model 2 is adjusted for PC 1 and PC2 score; [c] Model 3 is additionally adjusted for age, sex, asthma; [d] Model 3 is additionally adjusted for atopy; [e] Model 4 is additionally adjusted for WHO z-score for BMI.

Outcome		School		
	Walkability [β (95% CI)]	ICC	Variance	Explained variation*
**FVC**				
**Model 0**	-0·58 (-2·79; 1·63)	---	---	---
**Model 1[a]**	---	1·78%	4·48	Reference
**Model 2[b]**	-0·51 (-3·59; 2·56)	1·99%	5·04	-12·4%
**Model 3[c]**	-0·41 (-3·31; 2·49)	2·57%	5·20	-16·0%
**Model 4[e]**	-0·39 (-3·31; 2·54)	2·77%	5·49	-22·6%
**FEV_1**				
**Model 0**	-1·02 (-3·07; 1·02)	---	---	---
**Model 1[a]**	---	2·13%	4·53	Reference
**Model 2[b]**	-0·78 (-3·68; 2·12)	2·18%	4·76	-5·21%
**Model 3[c]**	-0·69 (-3·17; 1·79)	1·83%	3·36	25·8%
**Model 4[e]**	-0·66 (-3·20; 1·87)	1·94%	3·77	16·6%
**$FEF_{25\%-75\%}$**				
**Model 0**	-1·27 (-4·74; 1·98)	---	---	---
**Model 1[a]**	---	0·37%	2·14	Reference
**Model 2[b]**	-1·25 (-5·04; 2·53)	0·31%	1·80	15·7%
**Model 3[c]**	-1·29 (-4·79; 2·20)	2·31E-4%	1·27E-3	99·9%
**Model 4[e]**	-1·29 (-4·79; 2·20)	1·74E-4%	9·57E-4	99·9%
**EBC pH**				
**Model 0**	0·09 (-0·03; 0·21)	---	---	---
**Model 1[a]**	---	3·48%	0·03	Reference
**Model 2[b]**	0·07 (-0·13; 0·27)	3·54%	0·03	-1·94%
**Model 3[c]**	0·05 (-0·13; 0·24)	2·35%	0·02	33·0%
**Model 4[e]**	0·05 (-0·14; 0·24)	2·44%	0·02	30·5%
**Exhaled NO**				
**Model 0**	-0·05 (-0·17; 0·07)	---	---	---
**Model 1[a]**	---	3·98%	0·03	Reference
**Model 2[b]**	-0·03 (-0·24; 0·17)	4·34%	0·04	-9·46%
**Model 3[d]**	-0·07 (-0·29; 0·14)	4·85%	0·04	-17·8%
**Model 4[e]**	-0·07 (-0·29; 0·14)	4·97%	0·04	-20·7%
**Baseline pupil diameter**				
**Model 0**	-0·22 (-0·34; -0·09)	---	---	---
**Model 1[a]**	---	20·4%	0·158	Reference
**Model 2[b]**	-0·20 (-0·56; 0·16)	19·9%	0·154	2·85%
**Model 3[c]**	-0·18 (-0·55; 0·18)	19·6%	0·153	3·70%
**Model 4[e]**	-0·18 (-0·55; 0·18)	19·6%	0·153	3·71%
**Final pupil diameter**				
**Model 0**	-0·04 (-0·13; 0·05)	---	---	---
**Model 1[a]**	---	14·1%	0·053	Reference
**Model 2[b]**	-0·05 (-0·27; 0·18)	14·7%	0·056	-4·89%
**Model 3[c]**	-0·04 (-0·26; 0·18)	14·3%	0·055	-3·72%
**Model 4[e]**	-0·04 (-0·26; 0·18)	14·3%	0·055	-3·74%
**ACV**				
**Model 0**	-0·22 (-0·32; -0·12)	---	---	---
**Model 1[a]**	---	14·7%	0·070	Reference
**Model 2[b]**	-0·22 (-0·45; 0·01)	12·6%	0·058	16·6%
**Model 3[c]**	-0·20 (-0·43; 0·04)	12·8%	0·060	14·5%
**Model 4[e]**	-0·20 (-0·43; 0·04)	12·9%	0·060	14·1%
**MCV**				
**Model 0**	-0·31 (-0·45; -0·17)	---	---	---
**Model 1[a]**	---	12·3%	0·112	Reference
**Model 2[b]**	-0·31 (0·60; -0·01)	9·79%	0·088	21·3%
**Model 3[c]**	-0·27 (-0·56; 0·02)	8·95%	0·080	28·6%
**Model 4[e]**	-0·27 (-0·56; 0·02)	8·95%	0·080	28·6%
**Constriction amplitude**				
**Model 0**	1·94 (-2·65; -1·23)	---	---	---
**Model 1[a]**	---	8·38%	2·073	Reference
**Model 2[b]**	-1·83 (-3·00; -0·66)	4·38%	1·040	49·8%
**Model 3[c]**	-1·74 (-2·90; -0·59)	3·10%	0·729	64·8%
**Model 4[e]**	-1·74 (-2·90; -0·59)	3·12%	0·732	64·7%
**ADV**				

**Model 0**	0·01 (-0·04; 0·06)	---	---	---
**Model 1[a]**	---	8·57E-8%	8·10E-11	Reference
**Model 2[b]**	0·01 (-0·04; 0·06)	9·84E-8%	9·31E-11	-15·0%
**Model 3[c]**	0·01 (-0·05; 0·06)	1·04E-7%	1·01E-10	-24·5%
**Model 4[e]**	0·01 (-0·05; 0·06)	8·86E-8%	8·50E-11	-5·04%
**T75**				
**Model 0**	-0·17 (-0·29; -0·06)	---	---	---
**Model 1[a]**	---	4·53%	0·023	Reference
**Model 2[b]**	-0·17 (-0·34; -0·01)	3·21%	0·016	30·0%
**Model 3[c]**	-0·18 (-0·36; -0·01)	3·19%	0·016	29·2%
**Model 4[e]**	-0·18 (-0·36; -0·01)	3·19%	0·016	29·3%

Table S2. Multilevel model analysis of association between individual and walkability and lung function, pH, exhaled NO and pupillometry parameters explained by school.

*corresponds to the proportion of between-schools variance that could be explained by exposure and individual characteristics; 95% CI: 95% confidence interval; ICC: intra-class correlation coefficient; FVC: forced vital capacity; FEV_1: forced expiratory volume in the first second of FVC; $FEF_{25\%-75\%}$: forced expiratory flow in the middle portion of FVC; EBC: Exhaled breath condensate; ACV: Average constriction velocity; MCV: Maximum constriction velocity; ADV: Average dilation velocity; T75: the total time taken by the pupil to recover 75% of its initial resting diameter after it reached the peak of constriction.

Model 0 only included the PC1 and PC2 score; [a] Model 1 is null model, baseline model without any exposure variable; [b] Model 2 is adjusted for PC 1 and PC2 score; [c] Model 3 is additionally adjusted for age, sex, asthma; [d] Model 3 is additionally adjusted for atopy; [e] Model 4 is additionally adjusted for WHO z-score for BMI.

Land use classes	Median (IQR) m^2
Continuous Urban Fabric (S.L. > 80%)	289583·5 (201954·8)
Discontinuous Dense Urban Fabric (S.L.: 50% - 80%)	125793·8 (112971·6)
Discontinuous Medium Density Urban Fabric (S.L.: 30% - 50%)	12701·0 (16267·1)
Discontinuous Low Density Urban Fabric (S.L.: 10% - 30%)	0·0 (5874·0)
Discontinuous Very Low Density Urban Fabric (S.L. <30%)	0
Isolated Structures	0
Industrial, commercial, public, military and private units	139308·2 (105554·5)
Fast transit roads and associated land	0
Other roads and associated land	91338·1 (24958·3)
Railways and associated land	0·0 (0)
Port areas	0
Airports	0
Mineral extraction and dump sites	0
Construction sites	0·0 (5556·5)
Land without current use	4258·3 (10929·7)
Green urban areas	18918·6 (50231·3)
Sports and leisure facilities	8099·0 (13084·6)
Agricultural, Semi-natural areas, Wetlands	14611·4 (79938·4)
Forests	0·0 (0)
Water bodies	0·0 (0·)

Table S3. Characterization of neighborhood land use around evaluated schools.

IQR: interquartile range

Land uses classes	Components	
	PC1	PC2
Construction sites	-0·125	0·785*
Continuous Urban Fabric	-·0313	-0·732
Discontinuous Dense Urban Fabric	0·662*	0·335
Discontinuous Low Density Urban Fabric	0·369	0·139
Discontinuous Medium Density Urban Fabric	0·911*	0·016
Forests	0·021	0·238
Green urban areas	0·468*	-0·373
Industrial	-0·687	0·125
Land without current use	0·267	0·610*
Other roads	-0·263	0·166
Railways	-0·177	0·717*
Sports	0·020	-0·199
Water bodies	0·833*	0·080

Table S4. Rotated Component Matrix. * *Land uses classes which were included in each principal component*

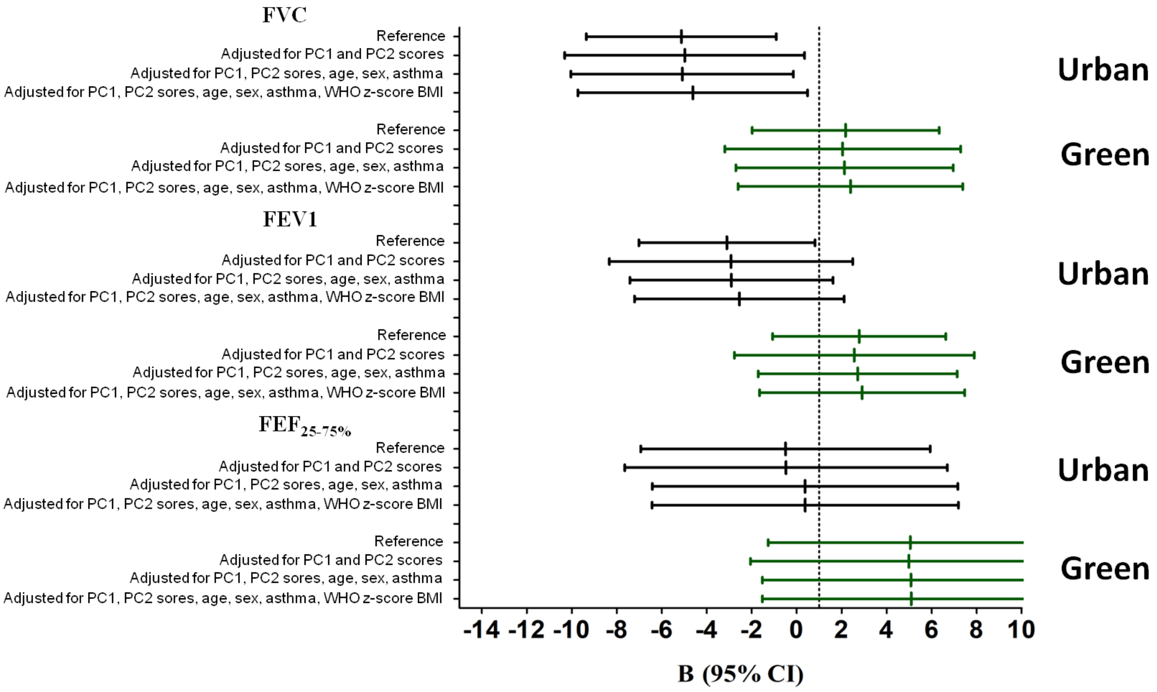

Figure S1. Association between neighborhood around school and lung function parameters.

Green lines correspond to PC1 (Discontinuous urban fabric, green urban areas and water bodies) and black lines correspond to PC2 (Construction sites, land without current use and railways). FVC: forced vital capacity; FEV_1: forced expiratory volume in the first second of FVC; $FEF_{25-75\%}$: forced expiratory flow in the middle portion of FVC; BMI: body mass index.

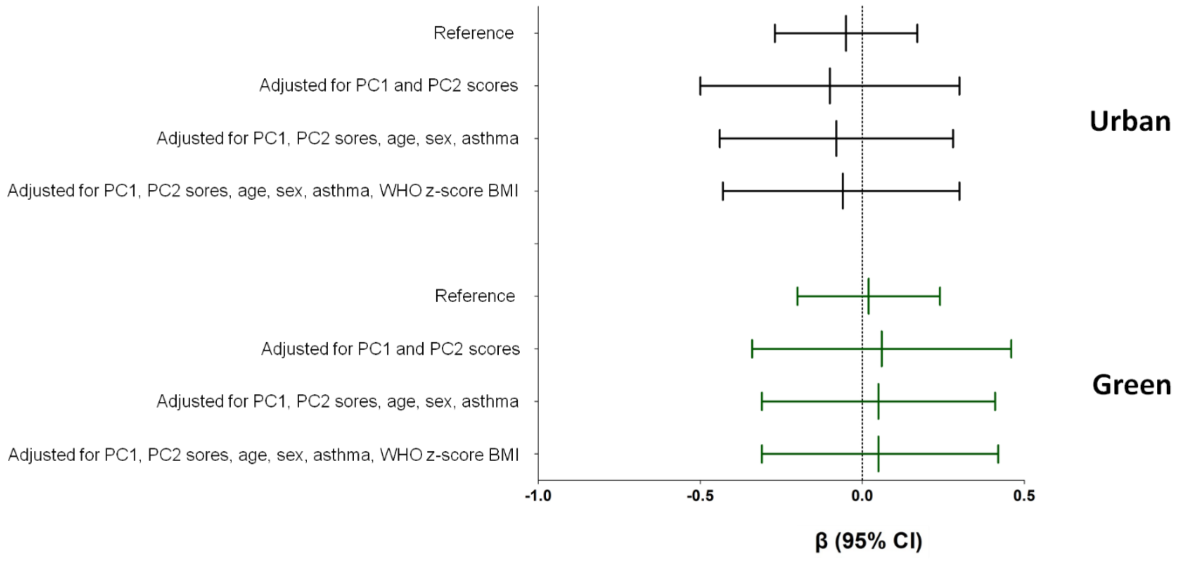

Figure S2. Association between neighborhood around school and EBC pH.

Green lines correspond to PC1 (Discontinuous urban fabric, green urban areas and water bodies) and black lines correspond to PC2 (Construction sites, land without current use and railways); BMI: body mass index.

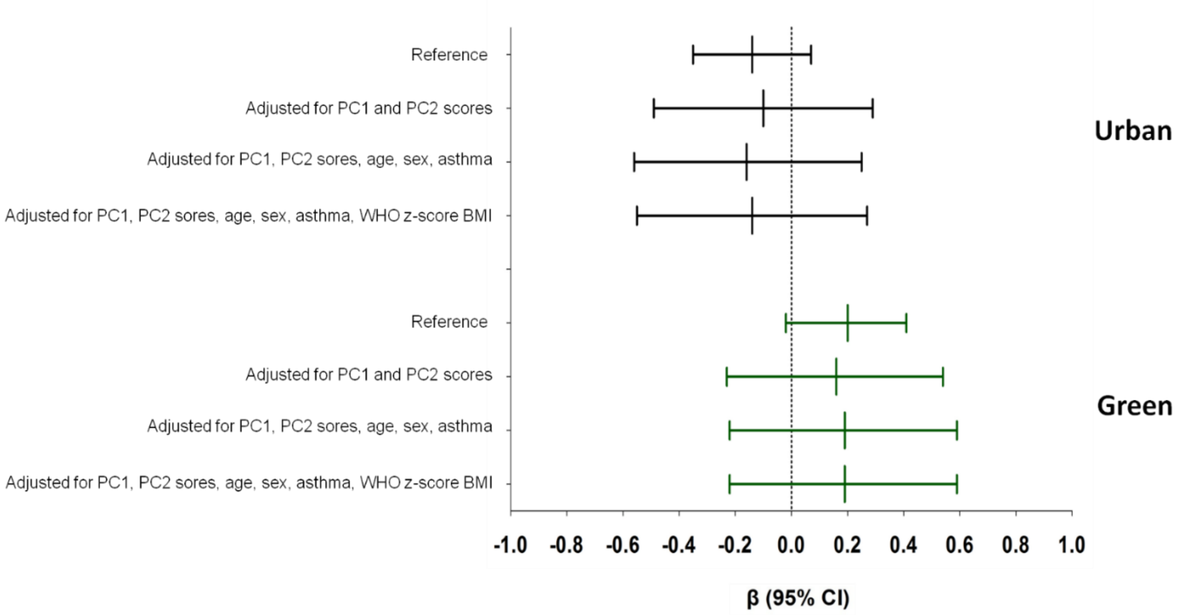

Figure S3. Association between neighborhood around school and exhaled level of nitric oxide.
Green lines correspond to PC1 (Discontinuous urban fabric, green urban areas and water bodies) and black lines correspond to PC2 (Construction sites, land without current use and railways); BMI: body mass index.

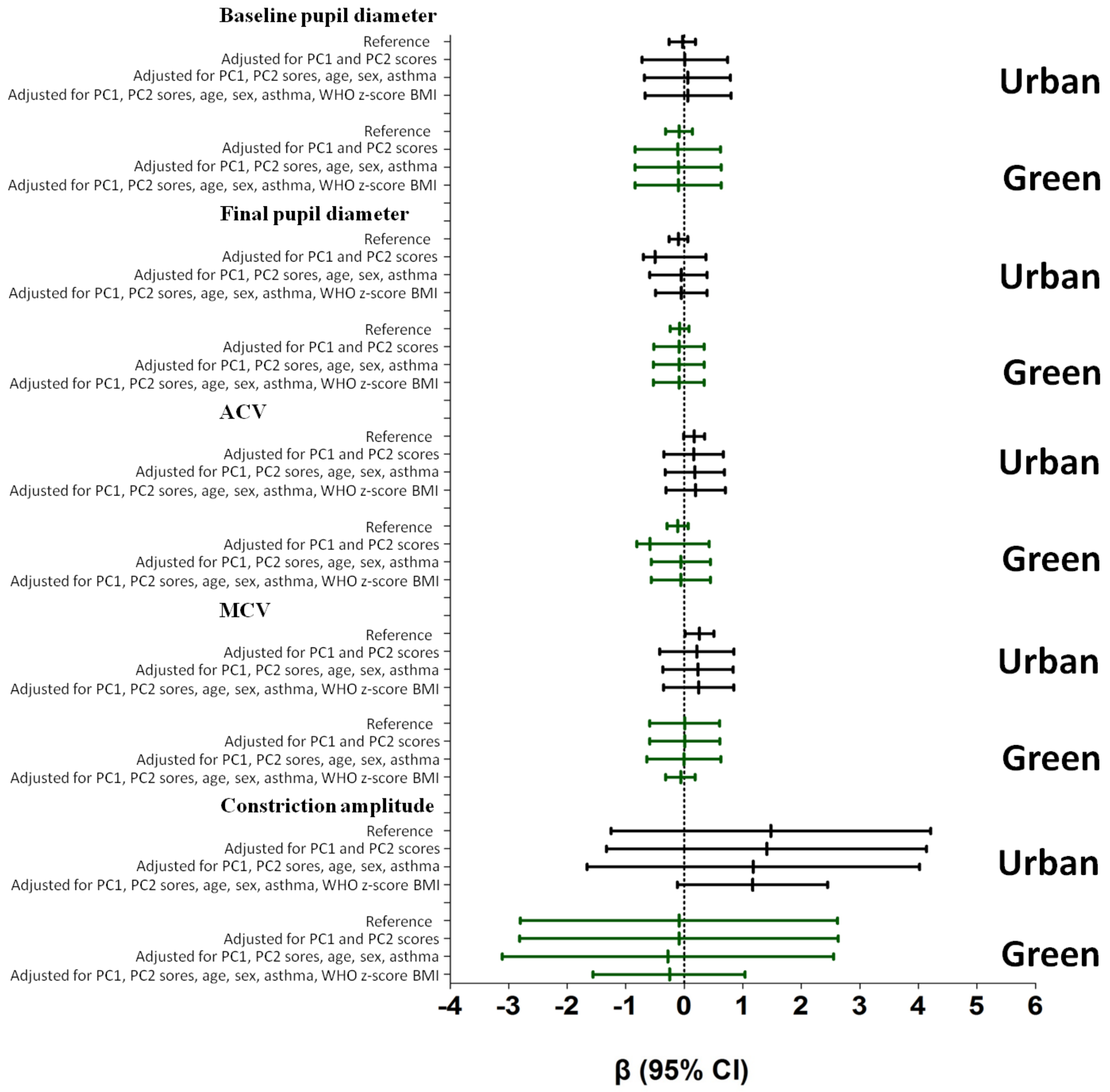

Figure S4a. Association between neighborhood around school and autonomic nervous system activity. Green lines correspond to PC1 (Discontinuous urban fabric, green urban areas and water bodies) and black lines correspond to PC2 (Construction sites, land without current use and railways). ACV: Average constriction velocity; MCV: Maximum constriction velocity; BMI: body mass index.

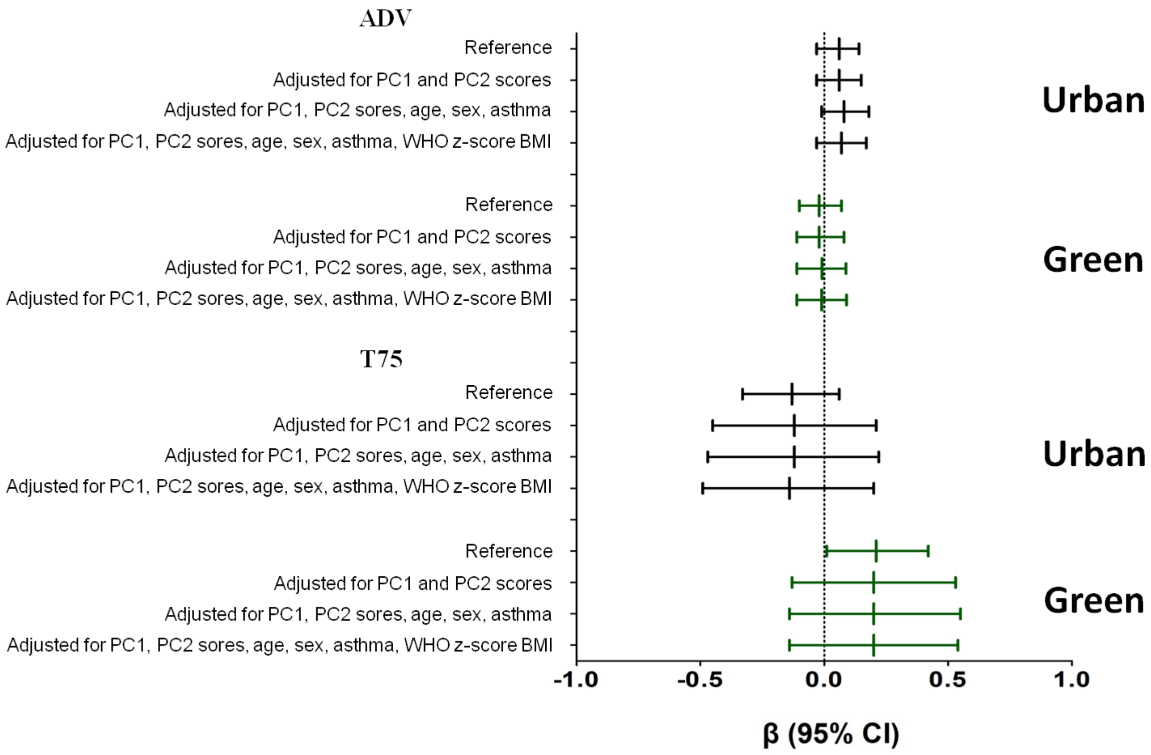

Figure S4b. Association between neighborhood around school and autonomic nervous system activity. Green lines correspond to PC1 (Discontinuous urban fabric, green urban areas and water bodies) and black lines correspond to PC2 (Construction sites, land without current use and railways). ADV: Average dilation velocity; T75: the total time taken by the pupil to recover 75% of its initial resting diameter after it reached the peak of constriction; BMI: body mass index.

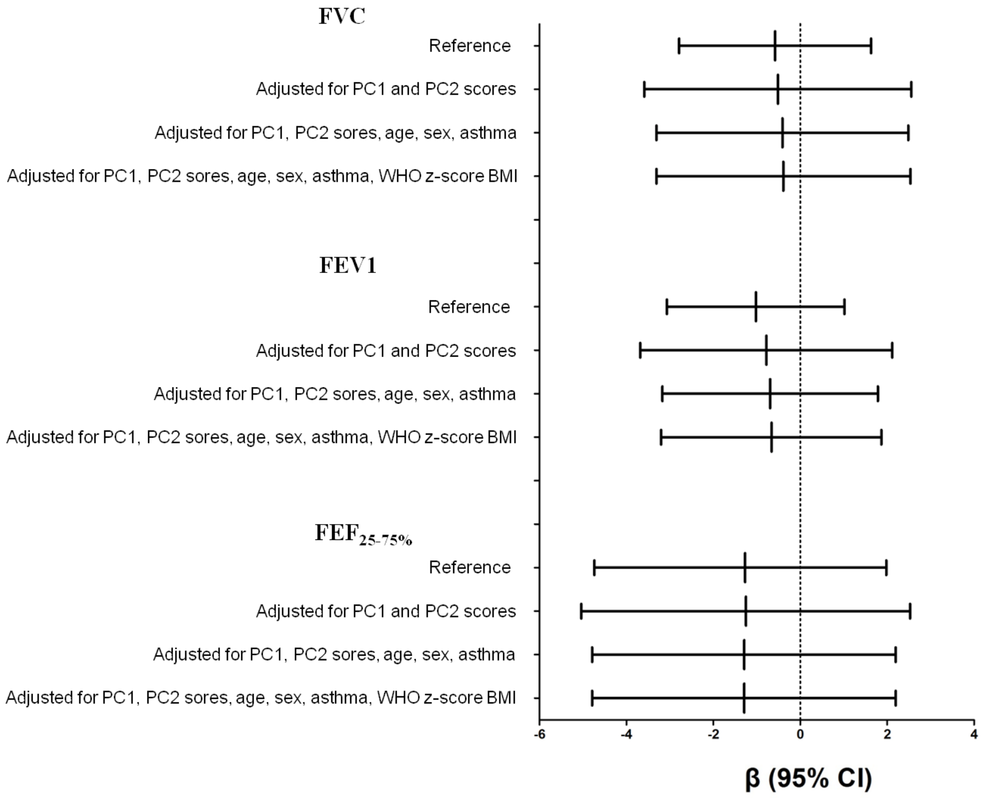

Figure S5. Association between walkability index around school and lung function parameters.

PC1: discontinuous dense urban fabric, discontinuous medium density urban land, green urban areas, and water bodies; PC2: construction sites, land without current use, and railways; FVC: forced vital capacity; FEV_1: forced expiratory volume in the first second of FVC; $FEF_{25\text{-}75\%}$: forced expiratory flow in the middle portion of FVC; BMI: body mass index.

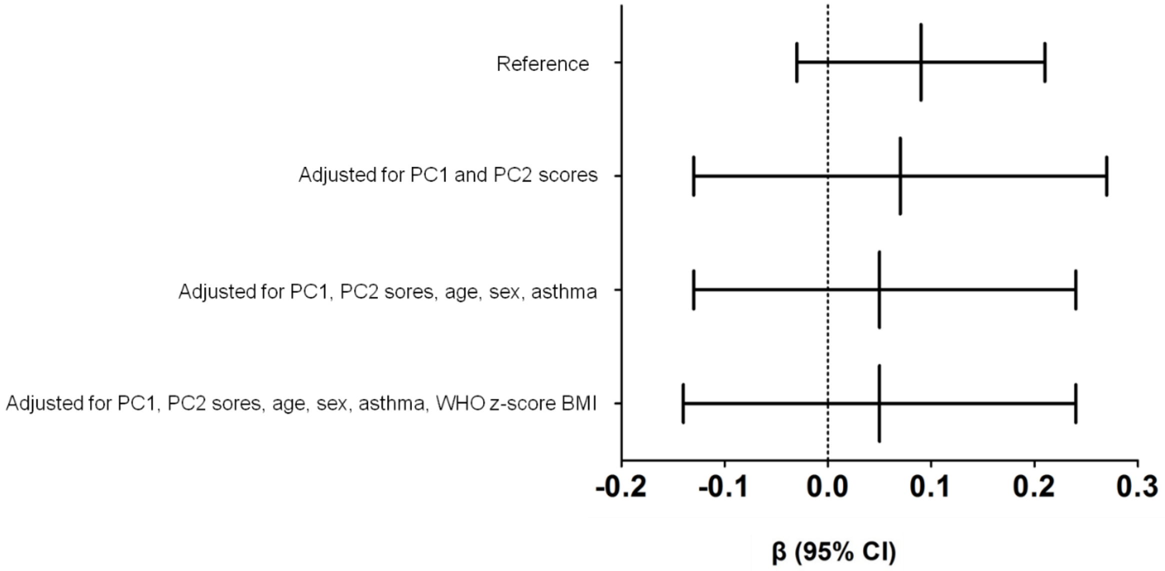

Figure S6. Association between walkability index around school and EBC pH.

PC1: discontinuous dense urban fabric, discontinuous medium density urban land, green urban areas, and water bodies; PC2: construction sites, land without current use, and railways; BMI: body mass index.

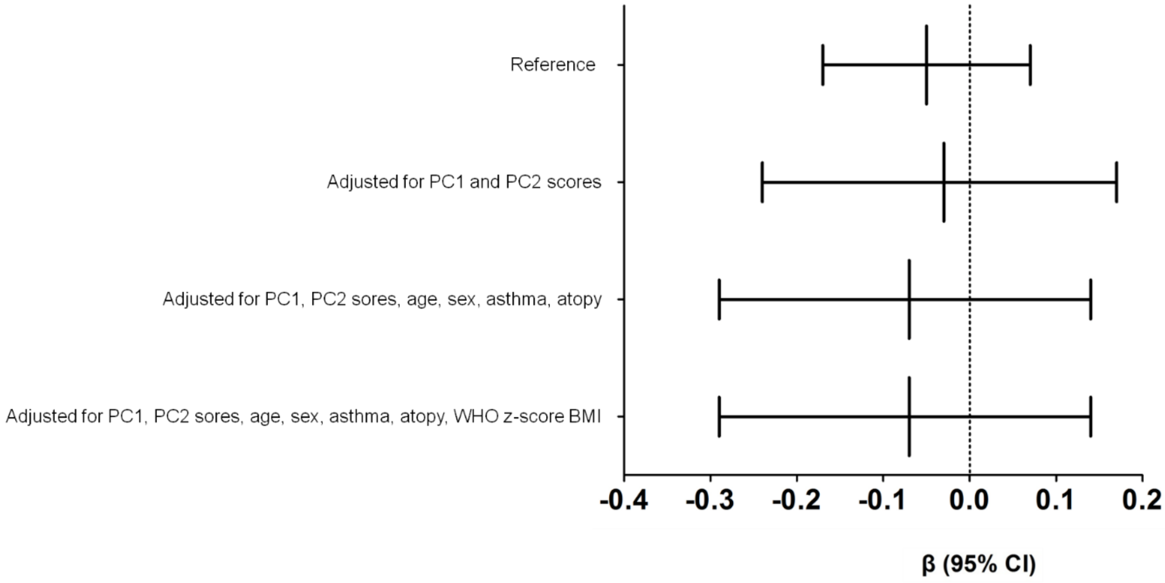

Figure S7. Association between walkability index around school and exhaled level of nitric oxide.
PC1: discontinuous dense urban fabric, discontinuous medium density urban land, green urban areas, and water bodies; PC2: construction sites, land without current use, and railways; BMI: body mass index.

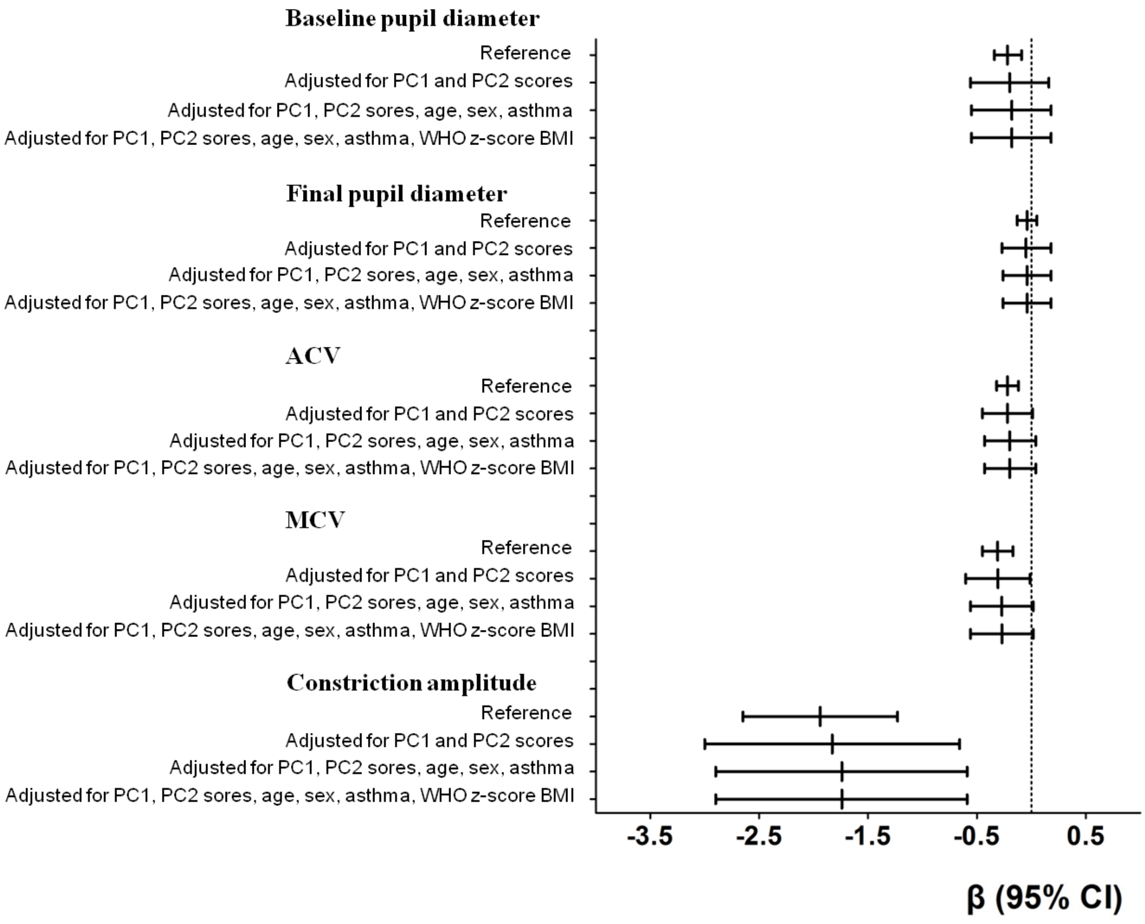

Figure S8a. Association between walkability index around school and autonomic nervous system activity. PC1: discontinuous dense urban fabric, discontinuous medium density urban land, green urban areas, and water bodies; PC2: construction sites, land without current use, and railways; ACV: Average constriction velocity; MCV: Maximum constriction velocity; BMI: body mass index.

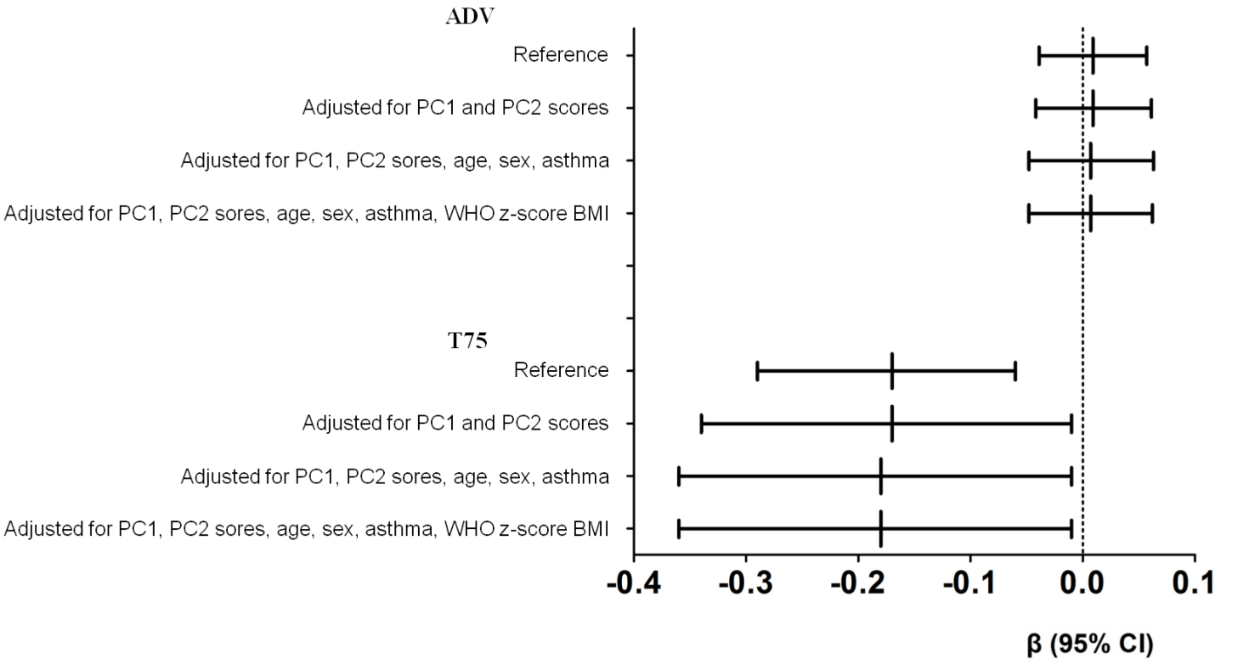

Figure S8b. Association between walkability index around school and autonomic nervous system activity. PC1: discontinuous dense urban fabric, discontinuous medium density urban land, green urban areas, and water bodies; PC2: construction sites, land without current use, and railways; ADV: Average dilation velocity; T75: the total time taken by the pupil to recover 75% of its initial resting diameter after it reached the peak of constriction; BMI: body mass index.

References

1. Weiland SK, Björkstén B, Brunekreef B, Cookson WOC, von Mutius E, Strachan DP. Phase II of the International Study of Asthma and Allergies in Childhood (ISAAC II): rationale and methods. *European Respiratory Journal* 2004; **24**(3): 406.
2. Madureira J, Paciencia I, Ramos E, et al. Children's Health and Indoor Air Quality in Primary Schools and Homes in Portugal-Study Design. *Journal of toxicology and environmental health Part A* 2015; **78**(13-14): 915-30.
3. Miller MR, Hankinson J, Brusasco V, et al. Standardisation of spirometry. *Eur Respir J* 2005; **26**(2): 319-38.
4. Quanjer PH, Stanojevic S, Cole TJ, et al. Multi-ethnic reference values for spirometry for the 3-95-yr age range: the global lung function 2012 equations. *Eur Respir J* 2012; **40**(6): 1324-43.
5. Stanojevic S. Global Lung Function Initiative. In: GLI-2012 Excel Sheet Calculator, editor. 4 ed; 2014.
6. Dweik RA, Boggs PB, Erzurum SC, et al. An official ATS clinical practice guideline: interpretation of exhaled nitric oxide levels (FENO) for clinical applications. *Am J Respir Crit Care Med* 2011; **184**(5): 602-15.
7. Horvath I, Hunt J, Barnes PJ, et al. Exhaled breath condensate: methodological recommendations and unresolved questions. *Eur Respir J* 2005; **26**(3): 523-48.
8. Prieto L, Ferrer A, Palop J, Domenech J, Llusar R, Rojas R. Differences in exhaled breath condensate pH measurements between samples obtained with two commercial devices. *Respir Med* 2007; **101**(8): 1715-20.
9. Vaughan J, Ngamtrakulpanit L, Pajewski TN, et al. Exhaled breath condensate pH is a robust and reproducible assay of airway acidity. *European Respiratory Journal* 2003; **22**(6): 889.
10. Borrill Z, Starkey C, Vestbo J, Singh D. Reproducibility of exhaled breath condensate pH in chronic obstructive pulmonary disease. *Eur Respir J* 2005; **25**(2): 269-74.
11. Kullmann T, Barta I, Horvath I. Effect of freezing on exhaled breath condensate pH. *Respir Med* 2007; **101**(12): 2566; author reply 7.
12. Kostikas K, Papatheodorou G, Ganas K, Psathakis K, Panagou P, Loukides S. pH in Expired Breath Condensate of Patients with Inflammatory Airway Diseases. *American Journal of Respiratory and Critical Care Medicine* 2002; **165**(10): 1364-70.
13. Hunt J. Exhaled breath condensate pH assays. *Immunol Allergy Clin* 2007; **27**(4): 597-+.
14. GINA. Global stategy for asthma management and prevention. 2015 ed; 2015.
15. Kuczmarski RJ, Ogden CL, Grummer-Strawn LM, et al. CDC growth charts: United States. *Adv Data* 2000; (314): 1-27.
16. Muppidi S, Adams-Huet B, Tajzoy E, et al. Dynamic pupillometry as an autonomic testing tool. *Clin Auton Res* 2013; **23**(6): 297-303.
17. Purves D, Augustine GJ, Fitzpatrick D, Hall WC, Lamantia AS, LE W. Principles of Cognitive Neuroscience: Sinauer Associates; 2012.
18. Wang Y, Zekveld AA, Naylor G, et al. Parasympathetic Nervous System Dysfunction, as Identified by Pupil Light Reflex, and Its Possible Connection to Hearing Impairment. *Plos One* 2016; **11**(4).
19. Milioni ALV, Nagy BV, Moura ALA, Zachi EC, Barboni MTS, Ventura DF. Neurotoxic impact of mercury on the central nervous system evaluated by neuropsychological tests and on the autonomic nervous system evaluated by dynamic pupillometry. *Neurotoxicology* 2017; **59**: 263-9.
20. Park HYL, Jung SH, Park SH, Park CK. Detecting autonomic dysfunction in patients with glaucoma using dynamic pupillometry. *Medicine* 2019; **98**(11).
21. van den Berg MMHE, Maas J, Muller R, et al. Autonomic Nervous System Responses to Viewing Green and Built Settings: Differentiating Between Sympathetic and Parasympathetic Activity. *International Journal of Environmental Research and Public Health* 2015; **12**(12): 15860-74.
